# Protein Kinase C-Mediated Hyperphosphorylation and Lateralization of Connexin 43 Are Involved in Autoimmune Myocarditis-Induced Prolongation of QRS Complex

**DOI:** 10.3389/fphys.2022.815301

**Published:** 2022-03-28

**Authors:** Chunlian Zhong, Huan Zhao, Xinwen Xie, Zhi Qi, Yumei Li, Lee Jia, Jinwei Zhang, Yusheng Lu

**Affiliations:** ^1^School of Material and Chemical Engineering, Minjiang University, Fuzhou, China; ^2^Fuzhou Institute of Oceanography, Fuzhou, China; ^3^State Key Laboratory of Molecular Vaccinology and Molecular Diagnostics, School of Life Sciences, National Institute of Diagnostics and Vaccine Development in Infectious Diseases, Xiamen University, Xiamen, China; ^4^Liancheng County General Hospital, Longyan, China; ^5^Department of Basic Medical Sciences, Medical College of Xiamen University, Xiamen, China; ^6^School of Basic Medicine, Gannan Medical University, Ganzhou, China; ^7^Xiamen Key Laboratory of Cardiovascular Disease, Xiamen Cardiovascular Hospital Xiamen University, Xiamen, China; ^8^Hatherly Laboratories, Medical School, College of Medicine and Health, Institute of Biomedical and Clinical Sciences, University of Exeter, Exeter, United Kingdom

**Keywords:** experimental autoimmune myocarditis, protein kinase C, connexin 43 channels, QRS duration, ion channels

## Abstract

Myocarditis is a serious and potentially life-threatening disease, which leads to cardiac dysfunction and sudden cardiac death. An increasing number of evidence suggests that myocarditis is also a malignant complication of coronavirus pneumonia, associated with heart failure and sudden cardiac death. Prolonged QRS complexes that are related to malignant arrhythmias caused by myocarditis significantly increase the risk of sudden cardiac death in patients. However, the molecular mechanisms are not fully known at present. In this study, we identify protein kinase C (PKC) as a new regulator of the QRS complex. In isolated hearts of normal rats, the PKC agonist, phorbol-12-myristate-13-acetate (PMA), induced prolongation of the QRS complex. Mechanistically, hyperphosphorylation and lateralization of connexin 43 (Cx43) by PKC induced depolymerization and internalization of Cx43 gap junction channels and prolongation of the QRS duration. Conversely, administration of the PKC inhibitor, Ro-32-0432, in experimental autoimmune myocarditis (EAM) rats after the most severe inflammation period still significantly rescued the stability of the Cx43 gap junction and alleviated prolongation of the QRS complex. Ro-32-0432 reduced phosphorylation and blocked translocation of Cx43 in EAM rat heart but did not regulate the mRNA expression level of ventricular ion channels and the other regulatory proteins, which indicates that the inhibition of PKC might have no protective effect on ion channels that generate ventricular action potential in EAM rats. These results suggest that the pharmacological inhibition of PKC ameliorates the prolongation of the QRS complex *via* suppression of Cx43 hyperphosphorylation, lateralization, and depolymerization of Cx43 gap junction channels in EAM rats, which provides a potential therapeutic strategy for myocarditis-induced arrhythmias.

## Introduction

Myocarditis is an inflammation disease of the heart muscle that is usually asymptomatic. Most hospitalized patients commonly present with infarction-like presentation and/or arrhythmias, and ventricular arrhythmias are fatal if they are not timely diagnosed and treated (Frustaci et al., 2019). Malignant arrhythmia is the most common cause of sudden cardiac death in patients with myocarditis, and effective therapeutic agents are urgently required. Cardiac electrical activity depends on well-controlled inward and outward ion channels, accessory proteins, and gap junction channels. On the surface electrocardiograms (ECGs), ventricular excitation is presented by the QRS complex. Prolonged QRS duration (≥ 120 ms) reflects intraventricular electrical dyssynchrony (Asano et al., 2019) and increases the risk of symptomatic arrhythmia (Ohkubo et al., 2011), which has become a significant independent predictor for cardiac death or heart transplantation in patients suspected to have myocarditis (Ukena et al., 2011). The patterns of QRS complex in myocardial hypertrophy could be potentially recognized for predicting ventricular arrhythmia, and the change in QRS complex patterns is closely associated with the density and distribution of Cx43 gap junctions (Bacharova, 2020).

Protein kinase C (PKC) isoforms, a family of serine–threonine kinases, mediate a wide range of signal-transduction pathways (Marrocco et al., 2019). The PKC family at least consists of 12 different isoenzymes. PKCα, ε, and ζ are expressed ubiquitously, whereas the other PKCs are tissue-specific. In fetal–neonatal hearts, PKCα, β, ε, and ζ levels are enriched relative to adult hearts (Goldberg and Steinberg, 1996). In the heart of mice, rats, humans, and rabbits, PKCα is the predominantly expressed PKC isoform (Hambleton et al., 2006). With respect to the heart, a number of studies have reported that PKC activation is associated with hypertrophy (Wang M. et al., 2020), dilated cardiomyopathy, ischemia–reperfusion injury (Hu et al., 2016), mitogen stimulation, and myocardial infarction (Wang et al., 2003). In pressure-overload cardiac hypertrophy and heart failure animal models, PKC-zeta activity is significantly increased (Li et al., 2019). Moreover, increased activity of PKCα, PKCβII, and PKCε has been reported in diabetic animal models (Min et al., 2017; Vetri et al., 2017). Consistently, transgenic mice with cardiac overexpression of PKCα showed decreased cardiac contractility (Braz et al., 2004). By contrast, short-term inhibition of conventional PKC isoforms significantly augments cardiac contractility and attenuates heart failure (Hambleton et al., 2006). In epithelial cells and fibroblasts, single-channel recording showed that the activation of PKC reduces the relative frequency of 100pS Cx43 channels and enhances the frequency of 51 pS low-conductance Cx43 channels (Lampe et al., 2000; Richards et al., 2004). Besides, we have reported that the heart of the EAM rat was enlarged, accompanied by increased expression of PKCα (Zhong et al., 2017). However, the mechanisms of PKC in ventricular electrical heterogeneity remain unknown.

In our previous work, we have demonstrated that experimental autoimmune myocarditis (EAM) rats are accompanied by prolonged QRS duration (Zhong et al., 2018) and an increase in PKC activity (Zhong et al., 2017). While numerous reports have confirmed a key role of PKC in heart contraction and Cx43 gap junctional communication (Rohr, 2004; Hambleton et al., 2006), less is known about the effect of PKC on ventricular electrical activity. Thus, we aim to investigate the relationship between PKC and QRS complex and the underlying molecular mechanisms in EAM rats.

## Materials and Methods

### Animals

Adult male Lewis rats (180–200 g) were purchased from Beijing Vital River Laboratory Animal Technology (Beijing, China). All animal care and experiments were performed in accordance with the procedures approved by the Animal Care and Use Committee of Minjiang University, which is in agreement with the 1975 Declaration of Helsinki, as revised in 2008.

### Induction of Experimental Autoimmune Myocarditis Model

Cardiac myosin was prepared from the ventricular muscle of porcine hearts by minor modifications to the method described in a previous study (Kodama et al., 1990; Zhong et al., 2018). Porcine hearts were obtained from landrace pigs from a selected abattoir. After the fat and vessels of the heart had been removed, the tissues were minced and washed three times. The minced tissue was then centrifuged for 5 min at 5,000 g. The pellet was gently stirred for 2 h in an equal volume of 0.3 mol/L KCl and 0.2 mol/L phosphate-buffered saline (PBS). The mixture was then centrifuged for 30 min at 10,000 g. The supernatant was filtered through 4 layers of gauze and precipitated with 9 volumes of cold water containing 1 mM EDTA, with vigorous stirring. After centrifuging at 10,000 g for 10 min, the pellet was dissolved in 25 mM Tris-HCl buffer (pH 7.5) containing 0.3 M KCl and 1 mM MgCl_2_. The solution was stirred gently overnight, and then, 2 mM ATP was added and stirred continuously for 20 min. The solution was obtained by centrifuging at 80,000 g for 3 h. The supernatant was diluted with 14 volumes of cold water containing 1 mM EDTA, left to stand for 30 min, and centrifuged at 10,000 g for 10 min. The pellet was suspended in 0.05 M pyrophosphate buffer (pH 7.5) containing 1 mM EDTA and 2 mM 2-mercaptoethanol and 2 mM ATP. Saturated ammonium sulfate solution adjusted to pH 7.0 was added, and a saturation of a fraction between 36 and 45% was collected. The cardiac myosin fraction was dissolved in potassium phosphate buffer, dialyzed overnight against potassium phosphate buffer, and the solution was centrifuged at 10,000 g for 30 min. The supernatant was freeze-dried and designated as cardiac myosin. All procedures were carried out at 4°C. To produce EAM, cardiac myosin was redissolved in a solution of 0.3 mol/L KCl and 0.2 mol/L PBS at a concentration of 10.0 mg/ml (Kodama et al., 1994; Watanabe et al., 2000; Chang et al., 2006, 2014). Each rat was immunized with 1 mg cardiac myosin in an equal volume of complete Freund’s adjuvant (CFA) supplemented with mycobacterium tuberculosis H37RA at a concentration of 10 mg/ml (Zhong et al., 2018). The rats of the control group were only immunized with CFA.

### *In vivo* Administration of Ro-32-0432 to Experimental Autoimmune Myocarditis Rats

Ro-32-0432 was dissolved in dimethylsulfoxide (DMSO) at a concentration of 3 mg/ml. It was intraperitoneally injected into rats every 2 days at a dosage of 1 mg/kg to the rats from days 14 to 18 of EAM. The rats in the control group were intraperitoneally injected with same volume of DMSO (Zhong et al., 2017).

### Preparation of Isolated Perfused Heart and Surface Electrocardiograms

Rats were anesthetized with isoflurane after treatment with heparin (10 mg/kg) for 30 min. The heart was quickly excised. After washing with the cold Krebs–Henseleit (KH) buffer solution, the heart was connected to Langendorff’s apparatus and perfused with modified Krebs–Henseleit solution. The modified KH buffer contains 118 mmol/L NaCl, 5.6 mmol/L KCl, 2.2 mmol/L CaCl_2_, 1.79 mmol/L NaHCO_3_, and 12.6 mmol/L glucose (pH 7.4), gassed with 95% O_2_ and 5% CO_2_. The heart was perfused at a constant pressure of 1,000 mm H_2_O and the temperature of 37.0 ± 0.5°C. The hearts were stabilized for 20 min, followed by perfusion with different solutions for 30 min. In control group, the isolated hearts were only perfused with modified KH buffer. The surface ECG was continuously recorded from the heart, with one electrode positioned at the base and one at the apex of the heart. Data collection and analysis were performed using a multichannel physiological signal recording system (BL-420S, Chengdu Techman Software Co., Ltd., China).

### Electrocardiography Recording

The ECG of normal and EAM rats was recorded under light isoflurane anesthesia (0.5%). The positive and the negative leads were tunneled to the left hindlimb and to the right shoulder, respectively. Data were analyzed using a BL-420S bio-experiment system (Chengdu Techman Software Co., Ltd., China). QRS duration was measured from the earliest to the latest deflection of the QRS complex, and its amplitude was measured from the nadir to the top of each QRS complex. QT interval was corrected according to the previous report: QTc = QT/(RR/100)1/2 (Mitchell et al., 1998).

### RNA Isolation and Real-Time PCR

Total RNA of the right and left cardiac ventricles was extracted using TRIzol reagent (Life technology, United States) following the manufacturer’s instructions. cDNA was synthesized from 3 μg of total RNA with a RevertAid First Strand cDNA Synthesis Kit (Thermo, Lithuania). The primers were synthesized by Takara Bio Inc. (Dalian, China) according to the cDNA sequences. Real-time PCR was carried out in the CFX96 Real-Time System using SYBR Green detection reagent (TOYOBO, Japan). The relative mRNA levels were calculated using the 2^–△△Ct^ method. Troponin T mRNA was used as the specific internal control for myocytes. The following gene-specific primer sequences were used for real-time PCR analysis: Nav1.5-forward, CAGCAGCTTCCGTAGGTTCA, Nav1.5-reverse, CATTGCCCAGGTCCACAAAT; Kv4.2-forward, CAACACTGGGGTATGGCGAC, Kv4.2-reverse, GCCACTT CCATGCAGCTTTC; Cav1.2-forward, CATCGAGGGTGAAAA CTGTG, Cav1.2-reverse, CATCACCAGCCAGTAGAAGA; RYR-forward, TCAATTTCCGCACCACCTAC, RYR-reverse, GAGCCAAAGATGAGCAGGTC; NCX-forward, GACCCAG AAGGAAATCAGAG, NCX-reverse, GAGACAAGCAATCGC AGACA; KChIP2-forward, GCACCTACGCTACTTTTCTC, KChIP2-reverse, TGATGCAGCCGTCCTTGTTG; SERCA2a-forward, CTATGACTGGTGATGGTGTG, SERCA2a-reverse, ATGGTGGAGAAGTTGTCGTC; Troponin T-forward, AGA GCGGAAGAGTGGGAAGA, Troponin T-reverse, GGCCTCT AGGTTGTGGATAC.

### Western Blot Analysis

All rats were sacrificed on day 21 of EAM. Total protein was prepared from the ventricle of rats. The cardiac ventricles were lysed in RIPA buffer (Product Code: P0013B, Beyotime, China) in the presence of 1 mmol/L phenylmethanesulfonyl fluoride (PMSF, Solarbio, China) and protein phosphatase inhibitor complex I (Aidlab, China), which contains sodium orthovanadate, sodium fluoride, sodium molybdate, sodium tartrate dihydrate, and imidazole. Protein samples were separated by 10% SDS-PAGE gels and were transferred to PVDF membranes (Millipore, United States). The membranes were blocked by 5% non-fat milk for 1 h and incubated with anti-connexin43 (1:10,000, Product Code: C6219, Sigma), anti-phospho-Cx43 (Ser368) (1:1,000, *Product Code: 3511*, Cell Signaling), anti-phospho-Cx43 (Ser262) (1:100, Product Code: sc-17219, Santa Cruz), anti-myristoylated alanine-rich C kinase substrate (MARCKS) (phospho S158) antibody (1:5,000, Product Code: ab81295, Abcam), and anti-GAPDH antibody (1:5,000, Epitomics). After rinsed with PBST for 5 times, the membranes were incubated with a goat anti-rabbit antibody (1:10,000, Sigma) for 1 h at room temperature. Immunoblots were developed using ChemiDoc*™* Imaging Systems (Bio-Rad). Densitometry of bands was analyzed using ImageJ software (National Institutes of Health, United States).

### Immunohistochemical Analysis

Heart samples were fixed in 10% formalin, embedded, and cut into 5-μm thick sections. After deparaffinization and rehydration, sections were placed into 3% H_2_O_2_ in methanol for 10 min to block endogenous peroxidase. For antigen retrieval, the sections were treated with citric acid buffer (pH 6.0) in a microwave oven for 15 min and then cooled for 30 min at room temperature. After rinsing with PBS, the sections were blocked with 10% bovine serum albumin for 30 min at room temperature, followed by overnight incubation with anti-connexin43 antibody (1:2,000, Sigma) and anti-phospho-Cx43 antibody (Ser368) (1:100, Cell Signaling) at 4°C. Afterward, the sections were rinsed with PBS, followed by incubation with horseradish peroxidase-conjugated secondary antibodies (1: 500, Jackson) for 1 h at room temperature. The immune reaction was performed using a DAB Kit (MXB, China) under the microscope. The sections were counterstained in hematoxylin, dehydrated in gradient alcohol, and mounted.

### Statistical Analysis

All data are expressed as mean ± standard deviation (SD). Non-parametric tests and one-way analysis of variance (ANOVA) were performed by using Prism 5.0 (GraphPad Software, Inc.) software. Two-way ANOVA for repeated measures and a *post-hoc* test for multiple comparisons were performed by SPSS software. *p* < 0.05 was considered significantly different.

## Results

### Protein Kinase C Agonist, Phorbol-12-Myristate-13-Acetate, Induces Abnormal QRS Complex (Prolonged Duration and Low Voltage) in Isolated Heart

**FIGURE 1:**
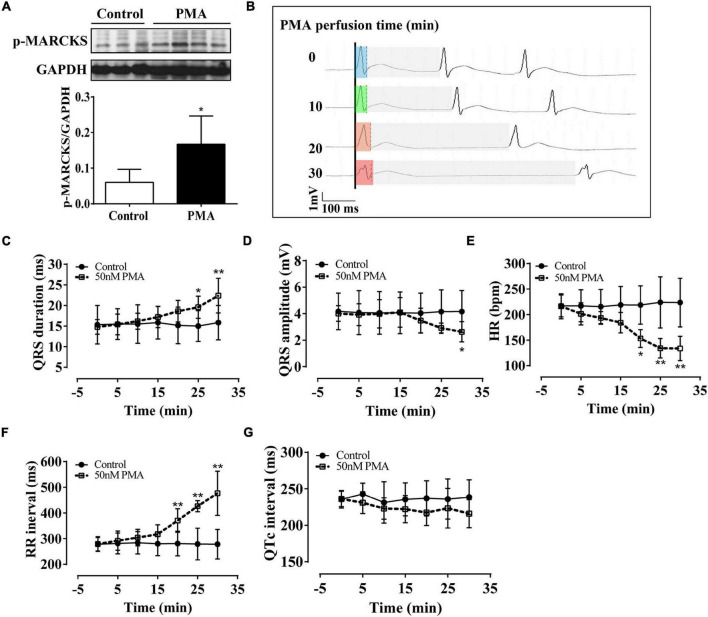
Phorbol-12-myristate-13-acetate (PMA) perfusion induced prolongation and low amplitude of the QRS complex in isolated normal rat hearts. The control hearts were perfused with the Krebs–Henseleit (KH) buffer solution. **(A)** Western blot analysis showing that PMA perfusion (50 nm) activated phospho-myristoylated alanine-rich C kinase substrate (MARCKS) protein. **(B)** Representative ECG recording in lead II showed QRS prolongation of the isolated hearts after PMA perfusion for 0, 10, 20, and 30 min. Light blue box: QRS duration of isolated heart after stabilization; light green box: QRS duration of isolated heart after 10-min of perfusion; light orange box: QRS duration of isolated heart after 20-min of perfusion; light red box: QRS duration of isolated heart after 30-min of perfusion; light gray box: RR interval. **(C–G)** Statistical data of electrocardiography (ECG), QRS duration **(C)**, QRS amplitude **(D)**, hear rate (HR) **(E)**, RR interval **(F)**, and QTc interval **(G)**. Error bars represent mean ± *SD*, *n* = 5 for each group. *p < 0.05 vs. control, **p < 0.01 vs. control.

To more directly ascertain the effect of PKC on ventricular electrical activity, we applied short-time (30 min) PMA (50 nm) infusion in Langendorff-perfused hearts of normal rats. Western blot analysis revealed that PMA significantly increased the phosphorylation of MARCKS protein at serine158, which has been used to demonstrate the activation of PKC (Figure 1A). Compared with the control group, short-time PMA infusion significantly widened QRS duration and induced low voltage of QRS complex (Figure 1B). Statistical data showed significant changes in the QRS complex after 20 min of PMA infusion (Figures 1C,D). Besides, PMA perfusion significantly decreased the HR (Figures 1E,F) but had no noticeable effect on QTc interval (Figure 1G). These data suggest that intraventricular electrical dyssynchrony is initiated by the activation of PKC, which mainly induces the QRS complex abnormality.

### Protein Kinase C Agonist, Phorbol-12-Myristate-13-Acetate, Induces the Hyperphosphorylation and Enhanced Lateralization of Connexin 43

**FIGURE 2:**
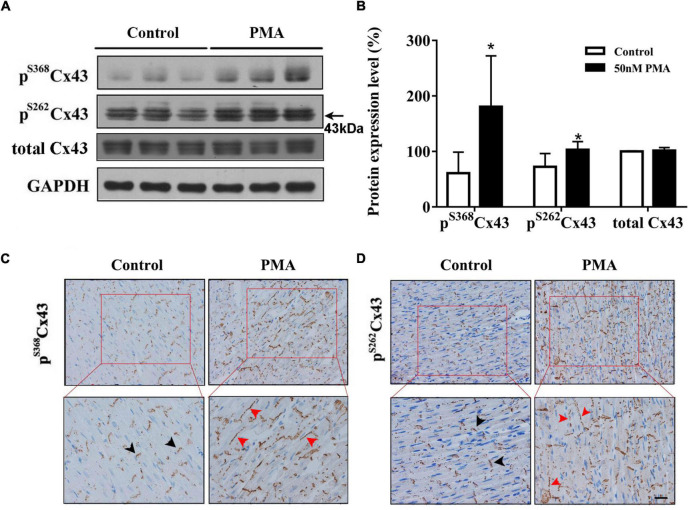
Short-time PMA perfusion increased phosphorylation of Cx43 at serine 368 and serine 262 and its redistribution in isolated hearts. **(A)** Western blot images showing pS368Cx43 and pS262Cx43 levels were increased after PMA perfusion (30 min). **(B)** Corresponding statistical data. **(C,D)** Immunohistochemical analysis of pS368Cx43 and pS262Cx43 expression and redistribution (Bar = 20 μm). The black arrows indicate normal Cx43 in the intercalated disc, and red arrows indicate Cx43 lateralization after PMA perfusion. Error bars represent mean ± *SD*, *n* = 3 for each group. *p < 0.05 vs. control (KH perfusion).

Gap junction channels play an important role in cardiac electrical conduction (Hoagland et al., 2019). In the ventricle, connexin43 (Cx43) is a principal gap junction channel protein. In our previous study, we have verified that hyperphosphorylated Cx43 at ser368 induces the prolongation of the QRS complex (Zhong et al., 2018). In this article, we study the effect of PKC on phosphorylation of Cx43. We found that phosphorylation levels of Cx43 at ser368 and ser262 were noticeably increased compared to controls (Figures 2A,B). Importantly, short-time infusion of PMA induced the relocation of pS262Cx43 and pS368Cx43 from intercalated disc to the lateral membranes of ventricular myocardium (Figures 2C,D). Moreover, abnormal patterns of pS262Cx43 and pS368Cx43 were observed, from dense to disperse. These data provide strong evidence that activation of PKC promotes the phosphorylation and lateralization of Cx43.

**FIGURE 3:**
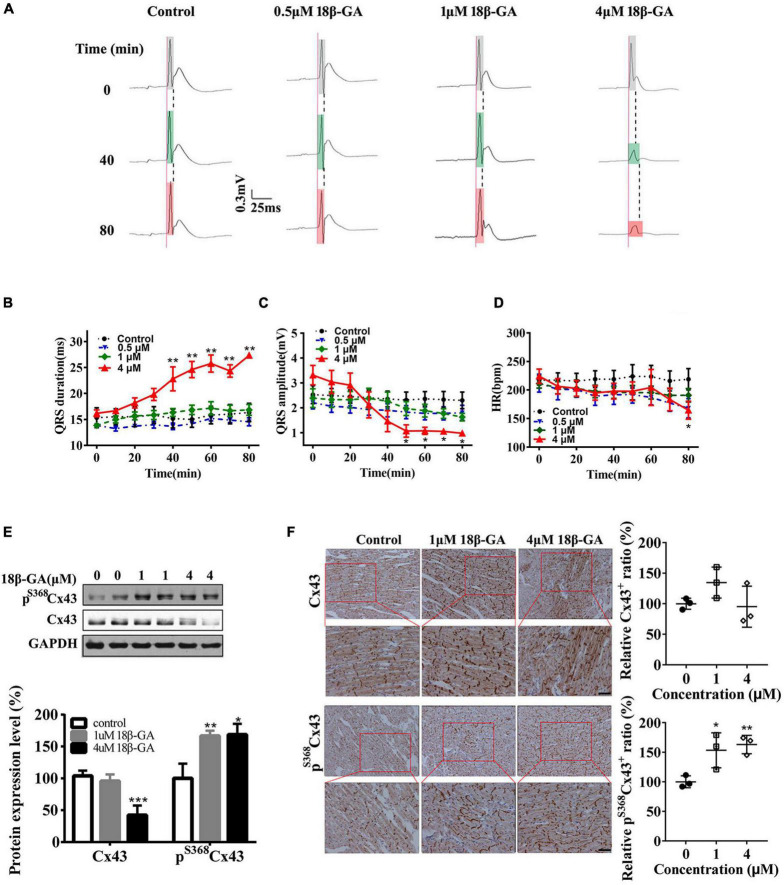
Cx43 hyperphosphorylation and lateralization induced by 18β-GA produce an abnormal QRS complex. **(A)** 18β-GA (0.5, 1, and 4 μm) perfusion (80 min) produced abnormal QRS duration of isolated rat hearts. Light gray box: QRS duration of isolated heart after stabilization; light green box: QRS duration of isolated heart after 40-min of perfusion; light red box: QRS duration of isolated heart after 80-min of perfusion. **(B–D)** Statistical data changes of QRS duration, QRS amplitude, and HR. *n* = 5 for each group. **(E)** Western blot images of pS368Cx43 and Cx43 in the control and 18β-GA-treated rats’ cardiac ventricles. The level of pS368Cx43 and Cx43 was normalized to GAPDH. **(F)** Immunohistochemical analysis of Cx43 and pS368Cx43 expression and redistribution (Bar = 20 μm), and corresponding statistical data of Cx43^+^ and pS368Cx43^+^ cells ratio. Error bars represent mean ± *SD*. *p < 0.05 vs. control; **p < 0.01 vs. control; ***p < 0.001 vs. control.

To further verify the relationship between Cx43 gap junction channels and the QRS complex, we perfused isolated hearts of normal rats with 18β-glycyrrhetinic acid (18β-GA) at different concentrations (0, 0.5, 1, and 4 μm) for 80 min, which effectively blocks the Cx43 gap junction (Yamamoto et al., 1998; Chung et al., 2007). Perfusion with 18β-GA gradually caused the prolonged duration and low amplitude of QRS complexes in a concentration-dependent manner (Figures 3A–C), along with a slight decrease in heart rate (HR) (Figure 3D). Furthermore, 18β-GA markedly decreased the total Cx43 expression (Figure 3E). Conversely, the phosphorylation of Cx43 at serine 368 in 1 and 4 μm 18β-GA perfusion group was markedly increased to 1.7-fold and 1.8-fold, respectively (Figure 3E). Consistent with western blot results, immunohistochemical analysis showed that the Cx43^+^ cells were decreased, whereas the pS368Cx43^+^ cells were significantly increased in the 4 μm 18β-GA perfusion group (Figure 3F). Moreover, 18β-GA induced the disassembly and diffusion of Cx43 and pS368Cx43 (Figure 3F). These data strongly suggest that the phosphorylation and pattern of Cx43 are the key regulators of the QRS complex.

### Protein Kinase C Inhibitor, Ro-32-0432, Prevents QRS Duration Prolongation and Low Voltage in Experimental Autoimmune Myocarditis Rat

**FIGURE 4:**
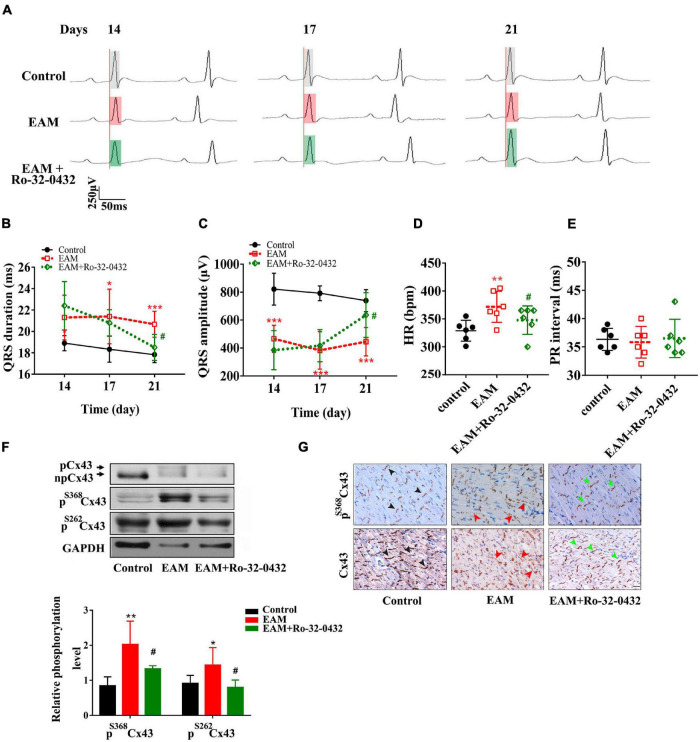
Blockade of protein kinase C (PKC) with Ro-32-0432 prevented the prolonged duration and low voltage of the QRS complex by suppressing Cx43 hyperphosphorylation and redistribution. **(A)** Representative ECG recording of normal, experimental autoimmune myocarditis (EAM), and Ro-32-0432-treated EAM rats. Each set of the ECG was recorded from the same rat on days 14, 17, and 21. Light gray box: QRS duration of control rats; light red box: QRS duration of EAM rats; light green box: QRS duration of Ro-32-0432-treated EAM rats. **(B–E)** Statistical data of ECG, QRS duration **(B)**, QRS amplitude **(C)**, HR **(D)**, and PR interval **(E)**. **(F)** Western blot images of pS368Cx43, pS262Cx43, and Cx43 in the cardiac ventricles of control, EAM, and Ro-32-0432-treated EAM rats. *n* = 6 for each group. **(G)** Immunohistochemical analysis of Cx43 and pS368Cx43 expression and redistribution (Bar = 20 μm). The black arrows in the pictures of the control group indicate the normal pattern of Cx43 and pS368Cx43 in intercalated disc; the red arrows indicate the lateralization of Cx43 and pS368Cx43 in the EAM group; the green arrows indicate partially recovered pattern and distribution of Cx43 and pS368Cx43 in Ro-32-0432-treated EAM group. Error bars represent mean ± *SD*. *p < 0.05 vs. control; **p < 0.01 vs. control; ***p < 0.001 vs. control; ^#^*p* < 0.05 vs. EAM.

To determine the role of PKC in ventricular electrical conduction, we applied bisindolylmaleimide compounds, Ro-32-0432, with certified selectivity and efficacy for PKC isoforms (Zhong et al., 2017) to prevent PKC activation. We administrated Ro-32-0432 (1 mg/kg) to EAM rats on day 14 after the most severity period, which was determined by cardiac function and histopathological evaluation (Supplementary Figure 1). On days 14, 17, and 21 of EAM, we recorded the ECG in lead II of rats in different groups. ECG measurements showed that QRS duration was significantly prolonged, whereas QRS amplitude was markedly decreased on day 14 of EAM (Figures 4A–C). The prolonged QRS duration was significantly shortened in Ro-32-0432-treated rats on day 17 of EAM (Figure 4B). On day 21 of EAM, Ro-32-0432 treatment totally inversed the prolongation of QRS duration from 22.4 ± 0.9 to 18.5 ± 0.5 ms (*p* < 0.05) (Figure 4B) and partially increased the QRS amplitude from 384.7 ± 57.0 to 636.0 ± 65.1 μv (*p* < 0.05) (Figure 4C). Concurrently, Ro-32-0432 exerted a protective effect on HR (Figure 4D). In contrast, PR interval showed no difference in the course of EAM (Figure 4E). Taken together, these data suggest that PKC is associated with ventricular conduction disorder, and inhibition of PKC activity suppresses the prolongation and low voltage of QRS complex.

### Protein Kinase C Inhibitor, Ro-32-0432, Downregulates the Phosphorylation of Connexin 43 and Prevents the Change in the Pattern and Distribution of Connexin 43 in Experimental Autoimmune Myocarditis Rat Hearts

Numerous studies have shown that PKC directly phosphorylates Cx43 at serine 368, which plays an important role in gap junctional trafficking, assembly, and/or degradation, and is related to molecular and electrical communication through gap junction channels (Bao et al., 2004). In this study, we showed that the phosphorylation of Cx43 at serine 368 was significantly decreased in Ro-32-0432-treated rats compared with EAM rats (Figure 4F). Moreover, we found that the phosphorylation of Cx43 at serine 262 in EAM was significantly increased to 1.6-fold of controls (*p* < 0.01) (Figure 4F). In Ro-32-0432-treated group, the phosphorylation of Cx43 at serine 262 was noticeably decreased compared to that in EAM rats (Figure 4F). These data support the involvement of PKC in the phosphorylation of Cx43 at serine 368 and serine 262, which are crucial in maintaining the gap junction communication.

Normal pattern and distribution of Cx43 are essential for electrical coupling in the myocardium, especially in the ventricular myocardium (Schultz et al., 2016). As shown in Figure 4G (middle panel), EAM induced dispersion pattern and lateralized distribution of Cx43. Conversely, administration of Ro-32-0432 restored the dense pattern and suppressed the lateralization of Cx43 (Figure 4G, right panel). In addition, Ro-32-0432 exerted protective effects on the pattern and localization of the phosphorylation of Cx43 at serine 368 (Figure 4G, upper panel).

### Protein Kinase C Inhibitor, Ro-32-0432, Has No Effect on the mRNA Level of Ventricular Ion Channels and the Other Regulatory Proteins

**FIGURE 5:**
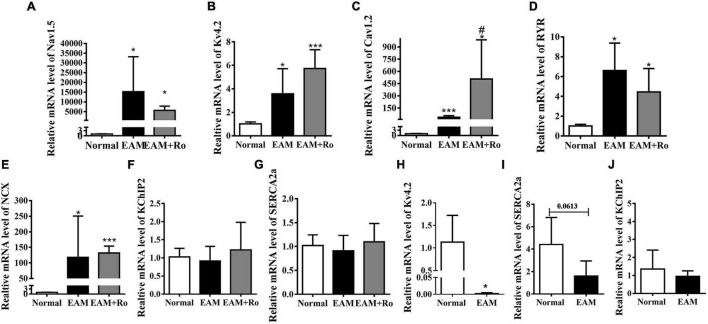
mRNA expression of ion channels and related regulatory proteins in normal, EAM, and Ro-32-0432-treated EAM rat hearts. **(A–G)** Relative mRNA expressions of Nav1.5, Kv4.2, Cav1.2, RYR, NCX, KchIP2, and SERCA2a. mRNA expressions of Kv4.2 and Cav1.2 were significantly increased in EAM rats, whereas Ro-32-0432 treatment did not have a significant reduction or even a slight increase (Cav1.2) compared with EAM rats. mRNA expressions of KchIP and SERCA2a had no difference in the hearts of normal, EAM, and Ro-32-0432-treated EAM rat. **(H–J)** mRNA expressions of Kv4.2, SERCA2a, and KchIP2 in the left ventricular wall. Error bars represent mean ± *SD*, *n* = 5 for each group. *p < 0.05 vs. control; ***p < 0.001 vs. control; ^#^*p* < 0.05 vs. EAM.

In ventricles, well-ordered electrical conduction depends on inward and outward ion channels, ion transporters, channel-regulating molecules, and gap junction channel proteins. We, therefore, investigated the expression levels of genes encoding predominant ion channels, ion transporters, calcium ATPase, and ion exchangers involved in generating ventricular action potential. The expression of Nav1.5 that generates main inward depolarization current in action potential was markedly increased in the ventricular tissues of EAM rats compared with those in the normal group (Figure 5A). Expressions levels of Kv4.2 encoding the major fraction of transient outward current, L-type Ca^2+^ channel, ryanodine receptors (RYRs) and Na^+^-Ca^2+^ exchanger (NCX), were increased to 3.5-fold, 38-fold, 6.6-fold, and 117-fold, respectively, in the myocardium of EAM rats (Figures 5B–E). K^+^ channel-interacting protein-2 (KChIP2) and sarcoplasmic reticulum Ca^2+^ ATPase (SERCA2a) reuptaking Ca^2+^ into sarcoplasmic reticulum were unchanged in the hearts of EAM rats (Figures 5F,G). However, the expression of Nav1.5, Kv4.2, KchIP2, SERCA2a, RYR, or NCX was unchanged or noticeably increased (L-type Ca^2+^ channel) in ventricular tissue of Ro-32-0432-treated EAM rats compared with EAM rats (Figures 5A–G). Furthermore, we examined the mRNA expression of Kv4.2, KchIP2, and SERCA2a in the left ventricular free wall. EAM induced significant suppression of Kv4.2, KchIP2, and SERCA2a mRNA expression in the left ventricular wall (Figures 5H–J), consistent with the previous reports (Wakisaka et al., 2004).

## Discussion

Myocarditis is commonly accompanied by severe complications, and arrhythmias especially in giant cell myocarditis is a main cause of cardiac sudden death in young patients. In this study, we used the EAM rat model closely resembling human giant cell myocarditis to investigate the effect of PKC on ventricular electrical activity and underlying molecular mechanisms. Our results are consistent with the earlier reports of abnormal QRS complex in patients with acute myocarditis and myocarditis animal models. In this study, we demonstrated that pharmacological inhibition of PKC with Ro-32-0432 could effectively suppress the depolymerization of Cx43 gap junction channels and ameliorate the prolongation of the QRS complex in EAM rat, which is closely associated with Cx43 phosphorylation and distribution. Our findings present a potential therapeutic strategy for the management of ventricular arrhythmias in myocarditis.

Cx43, a prominent cardiac gap junction protein, especially for ventricle, is essential to maintain cardiac function, including the development of heart (Boengler et al., 2021), and metabolic and electrical coupling (Beauchamp et al., 2012; De Smet et al., 2021). Stable electrical conduction *via* Cx43 gap junctions depends on the phosphorylation and distribution of constituent Cx43 proteins (van Veen et al., 2001). The hyperphosphorylation of Cx43 induces gap junction depolymerization and internalization (Solan and Lampe, 2014), and the lateralization of Cx43 impairs the cell–cell electrical coupling (Duffy, 2012), which leads to ventricular electrical conduction delay (prolonged QRS duration). The ablation of Cx43 leads to significant prolongation of QRS duration (Danik et al., 2004; Eckardt et al., 2006). In this study, we demonstrated that the hyperphosphorylation and lateralization of Cx43 are involved in the prolonged QRS complex in EAM rats, and blockade of PKC not only prevents the hyperphosphorylation and lateralization of Cx43 but also ameliorates the prolongation of QRS complex (Figure 4), which indicates that Cx43 may serve as a drug target for pharmaceutical intervention of myocarditis-associated with QRS complex.

Bisindolylmaleimides, Ro-32-0432, with a strong selectivity for PKC, especially PKCα (Wilkinson et al., 1993; Birchall et al., 1994), has recently been used as a therapeutic agent in many diseases. For example, Ro-32-0432 is used to treat T cell-mediated chronic inflammatory, autoimmune diseases (Birchall et al., 1994; Egu et al., 2019), heart failure (Hambleton et al., 2006), and tumor (Isakov, 2018). Moreover, Ro-32-0432 could be administrated in many ways, orally, intravenously, and intraperitoneally, which may provide therapeutics for a variety of poorly treated diseases. However, no specific and effective treatment options are available for myocarditis. Recently, the predictive role of ECG parameters has been investigated in patients with clinically suspected myocarditis and prolonged QRS duration acts as an independent predictor in cardiac death (Ukena et al., 2011; Marume et al., 2018). Therefore, effective amelioration of intraventricular electrical dyssynchrony is crucial for myocarditis therapy. In our previous studies, we have shown that 1 mg/kg dose of Ro-32-0432 by intraperitoneal administration significantly inhibited the activation of PKCα (Zhong et al., 2017). Here, we showed that the inhibition of PKC significantly ameliorates the prolongation of the QRS complex (Figures 4A,B).

The duration of the QRS complex is determined by the ventricular depolarization and the propagation of the excitatory cardiac impulse throughout the ventricle, which depends on inward and outward ion channels, ion transporters, channel-regulating molecules, and gap junction channel proteins (Kanno and Saffitz, 2001; Schram et al., 2002). Interestingly, PKC also regulates voltage-gated Na^+^ channel, Nav1.5, involved in the initiation and propagation of cardiac action potential. The downregulation of Nav1.5 slows the ventricular electrical conduction (Ning et al., 2016). The activation of PKC results in a voltage-dependent decrease in *I*_*Na*_ (Rook et al., 2012; Herren et al., 2013). In this study, we found that PKC significantly increased the Nav1.5 mRNA expression level in EAM rats (Figure 5A), which indicates that Nav 1.5 may not be a main contributor of the prolongation of QRS duration in the EAM rat model, since increased or decreased mRNA expression of the various channels does not equate to increased or decreased channel function. Therefore, to define the relationship of Nav1.5 channel and the prolonged QRS complex in EAM, the protein level and the function of Nav1.5 electrical conduction need further investigation. Interestingly, we previously reported that the heart of EAM rats was clearly enlarged and discolored, accompanied by increased expression and activity of PKCα but not the other PKC isoforms (Zhong et al., 2017). Noticeably, cardiac hypertrophy is associated with the changing QRS morphology (Bacharova, 2020). Fortunately, we further reveal the molecular mechanism of PKC regulating QRS complex morphology. Thus, we speculate that PKC could be a potential therapeutic strategy for cardiac hypertrophy. Taken together, our data strongly suggest that activation of PKC may produce an arrhythmogenic focus that may induce abnormal cardiac electrical activity, which results in malignant arrhythmias under disease conditions. This novel understanding of the cause of arrhythmia might lead to the concept that PKC inhibitor can be used as a new therapeutic for the arrhythmias in the acute phase of myocarditis.

In addition, myocarditis plays a vital role in coronavirus-induced pneumonia and increases the risk of sudden death. Development of myocarditis in numerous cases of coronavirus disease 2019 (COVID-19) has been reported (Siripanthong et al., 2020). For instance, among 138 patients showing severe symptoms of COVID-19, 44% had arrhythmia (Wang D. et al., 2020). However, the currently available antiviral treatments for COVID-19 may further aggravate arrhythmia (Wang D. et al., 2020). In this study, we have shown that inhibition of PKC ameliorates prolongation of the QRS complex, and further research will elucidate comprehensively the cardioprotective mechanisms of PKC inhibitors in the setting of inflammation. Our collective findings suggest that PKC could serve as a potential drug target for the treatment of patients with the COVID-19 infection through the suppression of arrhythmia.

## Conclusion

In our study, we demonstrated that pharmacological inhibition of PKC could effectively ameliorate the prolongation of the QRS complex in the EAM rat model, which is closely associated with Cx43 hyperphosphorylation and lateralization. This research provides a potential therapeutic strategy for the management of ventricular arrhythmias in myocarditis.

## Data Availability Statement

The raw data supporting the conclusions of this article will be made available by the authors, without undue reservation.

## Ethics Statement

The animal study was reviewed and approved by the Experimental Animal Ethics Committee of Minjiang University.

## Author Contributions

CZ: conceptualization. XX: methodology. HZ: software and project administration. XX and HZ: validation. XX and YML: formal analysis. CZ and XX: investigation. ZQ: resources and supervision. YSL: data curation and visualization. CZ and YSL: writing—original draft preparation. CZ, JZ, YSL, and LJ: writing, reviewing, editing, and funding acquisition. All authors have read and agreed to the published version of the manuscript.

## Conflict of Interest

The authors declare that the research was conducted in the absence of any commercial or financial relationships that could be construed as a potential conflict of interest.

## Publisher’s Note

All claims expressed in this article are solely those of the authors and do not necessarily represent those of their affiliated organizations, or those of the publisher, the editors and the reviewers. Any product that may be evaluated in this article, or claim that may be made by its manufacturer, is not guaranteed or endorsed by the publisher.
