# Supplementary material for: Protein Kinase C-Mediated Hyperphosphorylation and Lateralization of Connexin 43 Are Involved in Autoimmune Myocarditis-Induced Prolongation of QRS Complex
Source: Front Physiol. 2022 Mar 28;13:815301. doi: 10.3389/fphys.2022.815301 (PMC9000987; doi:10.3389/fphys.2022.815301)
Supplement: Supplementary file 1 [file Table_1.DOCX]

**Supplemental Materials**

**Protein Kinase C-Mediated Hyperphosphorylation and Lateralization of Connexin 43 Are Involved in Autoimmune Myocarditis-Induced Prolongation of QRS Complex**

Chunlian Zhong ^1,2^, Huan Zhao ^4^, Xinwen Xie ^5^, Zhi Qi ^6^, Yumei Li ^3^, Lee Jia ^1,2*^, Jinwei Zhang ^7,8*^, Yusheng Lu ^1,2*^

^1^School of Material and Chemical Engineering, Minjiang University, Fuzhou, Fujian 350108, China

^2^ Fuzhou Institute of Oceanography, Fuzhou, Fujian 350004, China

^3^ School of Basic Medicine, Gannan Medical University, Ganzhou, Jiangxi 341000, China

^4^State Key Laboratory of Molecular Vaccinology and Molecular Diagnostics, National Institute of Diagnostics and Vaccine Development in Infectious Diseases, School of Life Sciences, Xiamen Uni-versity, Xiamen 361102, China

^5^Liancheng county general hospital, LongYan, Fujian 366200, China

^6^ Department of Basic Medical Sciences, Medical College of Xiamen University, Xiang’an Nan Lu, Xiamen 361102, China

^7^Xiamen Key Laboratory of Cardiovascular Disease, Xiamen Cardiovascular Hospital Xiamen Uni-versity, Xiang’an Nan Lu, Xiamen 361102, China

^8^Institute of Biomedical and Clinical Sciences, Medical School, College of Medicine and Health, University of Exeter, Hatherly Laboratories, Exeter EX4 4PS, UK

*** Correspondence:**

Lee Jia, Jinwei Zhang and Yusheng Lu
lu_yu_sheng@126.com or 2622@mju.edu.cn (YL); j.zhang5@exeter.ac.uk (JZ); pharmlink@gmail.com or cmapcjia1234@163.com (LJ)


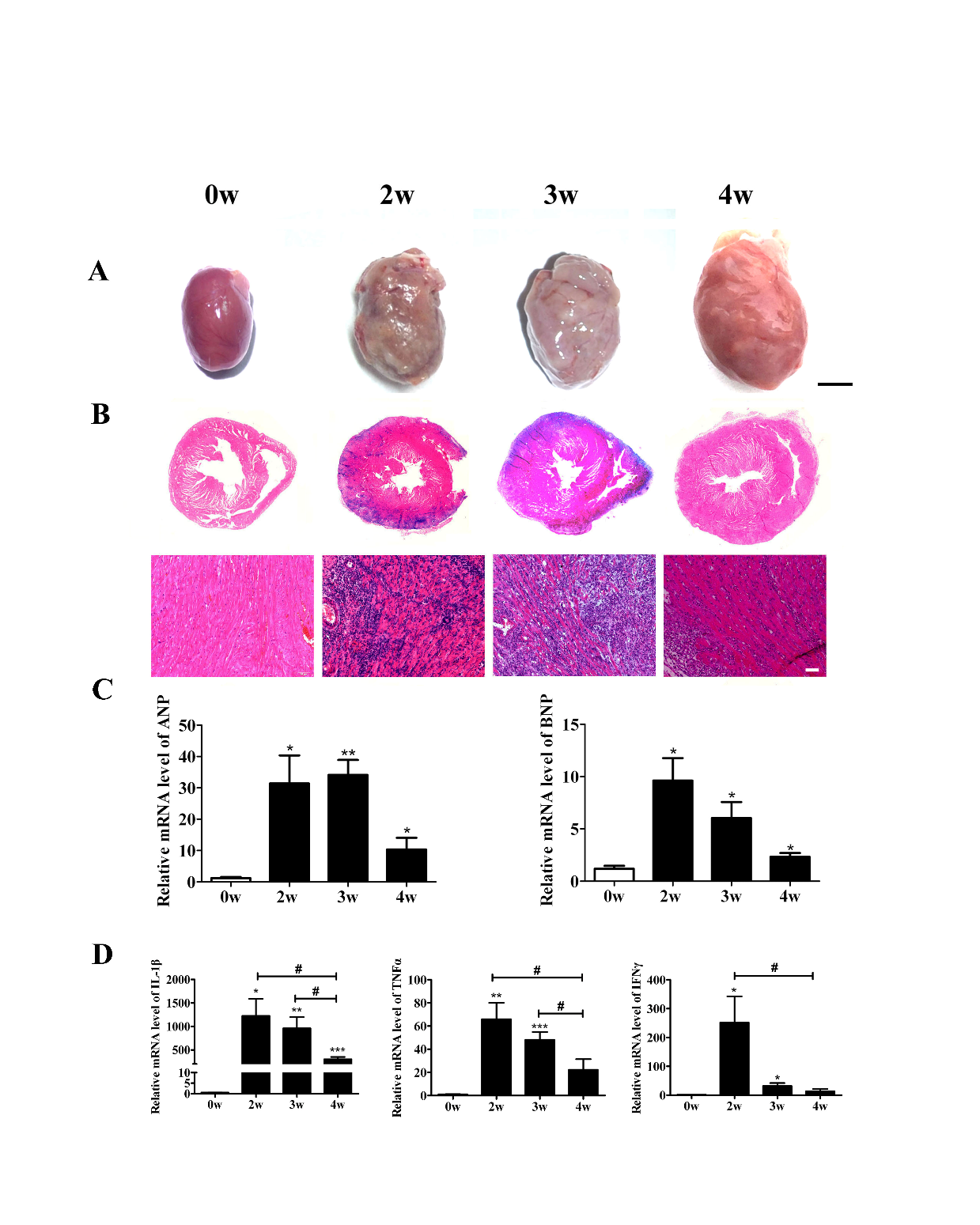


**Supplementary Figure 1.** Histopathological and cardiac function examination in the time course of EAM. 32 rats were immunized with cardiac myosin in CFA supplemented with mycobacterium tuberculosis H37RA, and then were sacrificed on the 14th, 21th and 28th day. Six control rats were sacrificed on Day 28. Hearts were obtained for pathological evaluation and mRNA examination. (**A**) Representative heart images of EAM rat on day 14, 21 and 28 (Bar = 5mm). (**B**) HE staining of ventricular myocardium (Bar = 50μm). (**C-D**) Relative mRNA level of ANP, BNP (**C**) and IL-1β, TNF-α and IFN-γ (**D**). ANP, atrial natriuretic peptide; BNP, B-type natriuretic peptide. n=6 for each group. *: p<0.05, **: p<0.01, ***: p<0.001*vs*. 0w；#: p<0.05, ##: p<0.01*vs*. 4w.
